# Supplementary material for: Cognitive, emotional, and social functioning of preschoolers with attention deficit hyperactivity problems
Source: BMC Psychiatry. 2022 Feb 1;22:78. doi: 10.1186/s12888-021-03638-9 (PMC8808769; doi:10.1186/s12888-021-03638-9)
Supplement: Supplementary file 1 — Additional file 1. [file 12888_2021_3638_MOESM1_ESM.pdf]

Supplementary Material A

## Recruitment

Figure S1 desribes the recruitment and inclusion procedure.

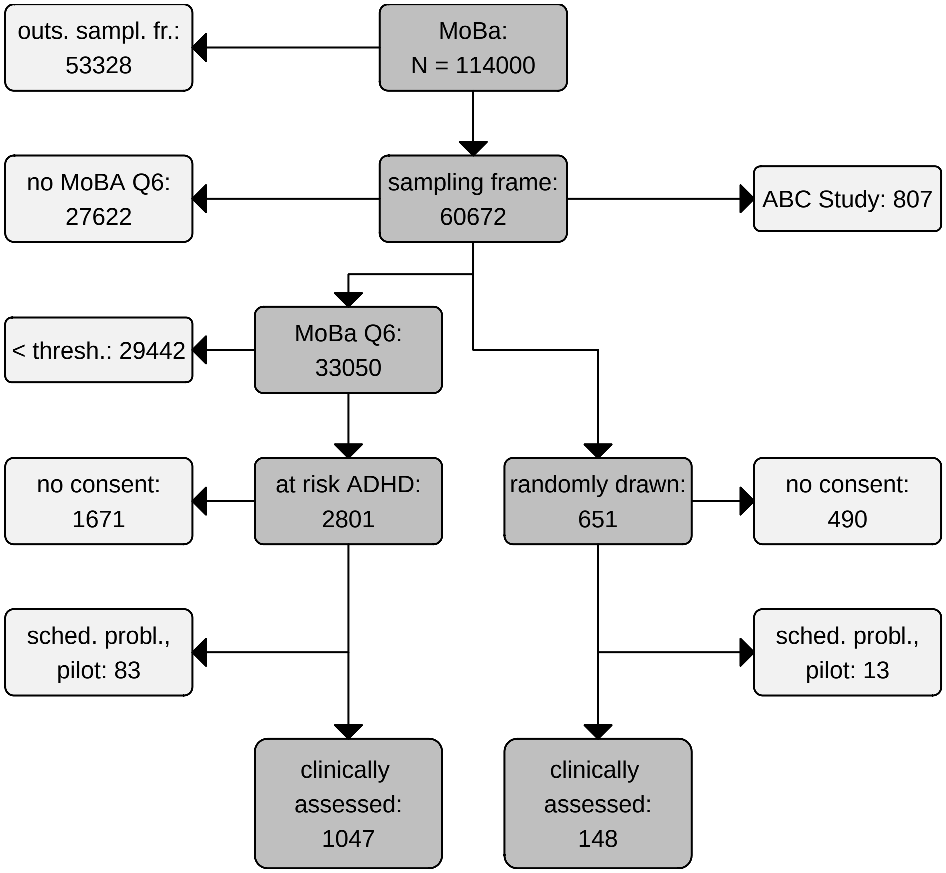


*Figure S1*. Recruitment flow chart. The inclusion period for the ADHD study lasted from April 2004 to January 2008. Q6 is the MoBa questionnaire sent out at child age three years. Participants of the ABC Study [1] were not eligible for the ADHD Study. "thresh." is the cut-off sum-score for identification of preschoolers at risk for ADHD. Some participants who consented participated in pilot assessments, and for some there was no assessment due to scheduling problems.

### ADHD items in the Moba questionnaire for 3 year olds

Items taken from the Child Behavior Checklist are indicated by [CBCL] and items derived from DSM-4 symptoms for ADHD are indicate by [DSM-4].

- Can't concentrate, can't pay attention for long [CBCL]
- Can't sit still, restless or overactive [CBCL]
- Can't stand waiting, wants everything now [CBCL]
- Demands must be met immediately [CBCL]
- Gets into everything [CBCL]
- Quickly shifts from one activity to another [CBCL]
- Becomes distracted or diverted by outside stimuli (sounds or events) [DSM-4]
- Finds it difficult waiting his/her turn [DSM-4]
- Has problems keeping focused on tasks or activities [DSM-4]
- Is excessively talkative [DSM-4]
- Doesn't seem to listen when he/she is being spoken to [DSM-4]

### Full list of instruments

The ADHD study used the semi-structured clinical interview "Preschool Age Psychiatric Assessment" [PAPA; [2]] to assess mental health symptoms. The PAPA interview elicits, based on DSM IV diagnostic classifications, information about the severity and frequency of symptoms and impairment for many mental health disorders of the childhood including ADHD, conduct disorder, oppositional defiant disorder, and anxiety disorders.

Parents and kindergarten teachers filled out the following questionnaires:

- Strength and Difficulties Questionnaire [SDQ, used from 2007-2009, 3]
- Conners' Rating Scale – Revised, Short version, for parents [CPRS-RS, used from 2007-2009, 4]

- Early Childhood Inventory 4 [ECI4, used from 2010-2012; [5]]
- Behavior Rating Inventory of Executive Function – preschool version [BRIEF-P, 6, 7]
- and the language section of the Child Development Inventory [CDI, 8, 9].

In addition, only parents received the

- Children's Behavior Questionnaire [CBQ, 10]
- Emotionality, Activity and Shyness temperament questionnaire [EAS, 11, 12]
- and also responded again to the 11 screening questions used to detect ADHD cases.

Only teachers received the Preschool Play Behavior Scale [PPBS, 13].

To facilitate cooperation with a sister-study, some questionnaire instruments were exchanged half way through the study. Until 2009 parents and teachers received the SDQ and the CPRS-RS and from 2010 on the ECI-4 and some impairment questions from the SDQ. Around the same time, the wording of few BRIEF-P items were updated, which had however no effect on the scale performance [14].

As neuropsychological tests the ADHD study employed the

- Developmental NEuroPSYchological Assessment Battery [15, 16],
- Spin The Pots and Truck Reversal Learning tests [17]
- Grooved Pegboard Task from the Wisconsin motor steadiness battery [18],
- Cookie Delay Task,
- Norwegian version of the Boston Naming test [BNT, 19].
- From the Stanford-Binet Intelligence Scales 5th ed. [20] the ADHD study used the Verbal Memory for Sentences test (VMS) to assess verbal working memory (VWM), the Object series, Pattern analysis/Object matrices tests (OS/PAM) to assess nonverbal IQ (NVIQ), the Comprehension/Vocabulary tests (CM) to assess verbal IQ (VIQ), and a task for verbal task fluid reasoning (VOS).

**Description of Cookie Delay Task.** The Cookie delay task [21] involved placing a cookie under one of several upturned transparent cups. The child was instructed to wait for a signal (clap) before retrieving the treat. Eight trials were given in a pseudo-random order with delays of between 5 and 30 seconds. The experimenter's hands were raised at the midpoint of the delay period ready to clap. The child was given one practice trial. The scoring was 0 - not inhibited, 1- partially inhibited, and 2 - fully inhibited, the total range was 0-16.

## Supplementary methods

All analysis were performed with R [Version 4.0.2; [22]][1] or Mplus [55]. Scripts for all analysis steps are available at https://github.com/gbiele/compfunc.

### Classification of mental health problems.

#### *Criteria for diagnostic classification.*

Mental health problems were classified according to DSM IV symptom criteria. In particular, criteria are

- for ADHD: At least 6 symptoms in either the inattention or hyperactivity-impulsivity domain

- for behavior problems: (a) Conduct Disorder: At least three symptoms, (b)

[1] We, furthermore, used the R-packages *arsenal* [Version 3.6.2; [23]], *brms* [Version 2.15.0; [24]; [25]], *car* [Version 3.0.10; [26]; [27]], *carData* [Version 3.0.4; [27]], *data.table* [Version 1.14.0; [28]], *diagram* [Version 1.6.5; [29]], *flextable* [Version 0.6.3; [30]], *ggplot2* [Version 3.3.5; [31]], *Gmisc* [Version 2.0.1; [32]], *haven* [Version 2.3.1; [33]], *HDInterval* [Version 0.2.2; [34]], *htmlTable* [Version 2.1.0; [35]], *knitr* [Version 1.33; [36]], *lattice* [Version 0.20.41; [37]], *mice* [Version 3.13.0; [38]], *MplusAutomation* [Version 0.8; [39]], *officer* [Version 0.3.16; [40]], *papaja* [Version 0.1.0.9997; [41]], *plotrix* [Version 3.8.1; [42]], *psych* [Version 2.0.12; [43]], *RColorBrewer* [Version 1.1.2; [44]], *Rcpp* [Version 1.0.7; [45]; [46]], *reshape* [Version 0.8.8; [47]], *rstanarm* [Version 2.21.1; [48]], *shape* [Version 1.4.5; [47]; [49]], *sjPlot* [Version 2.8.7; [50]], *StanHeaders* [Version 2.26.1; [51]], *stringi* [Version 1.7.3; [52]], *stringr* [Version 1.4.0; [53]], and *tableone* [Version 0.12.0; [54]].

Oppositional defiant disorder: At least four symptoms.

- for anxiety problems: (a) Specific phobia: At least one symptom, (b) social phobia: At least one symptom, (c) separation anxiety: At lest three symptoms (d) “Generalized Anxiety, worries” A strong worries combined with physical symptoms

We further distinguished children with and without impairments, based on a section about impairments the ADHD Study had added to each Disorder section of the PAPA manual.

*Assessment of impairment.*

For each mental health problem, impairments were assessed with questions about the impact of the symptoms on:

- the child’s ability to get along with the parents an the rest of the family
- the child’s ability to keep friends
- the child’s ability to learn or work in kindergarten
- the child’s ability to participate in play or other activities outside the kindergarten
- the child’s quality of life
- the family (is the child a burden for the parents or the rest of the family)

Each impairment was assessed on a scale from 0-3: “not at all”" (score 0), “a bit” (1), “a good deal” (2) , “a lot” (3).

Symptoms were classified as impairing if at least 2 questions were scored with 1, or if at least 1 questions was scored with 2 or higher.

The PAPA interview does not have a section for language impairments. However, in the ADHD Study clinicians evaluated language problems (Expressive language problems, combined expressive and phonological problems, phonological problems) as clinical (with impairment) or sub-clinical (without impairment).

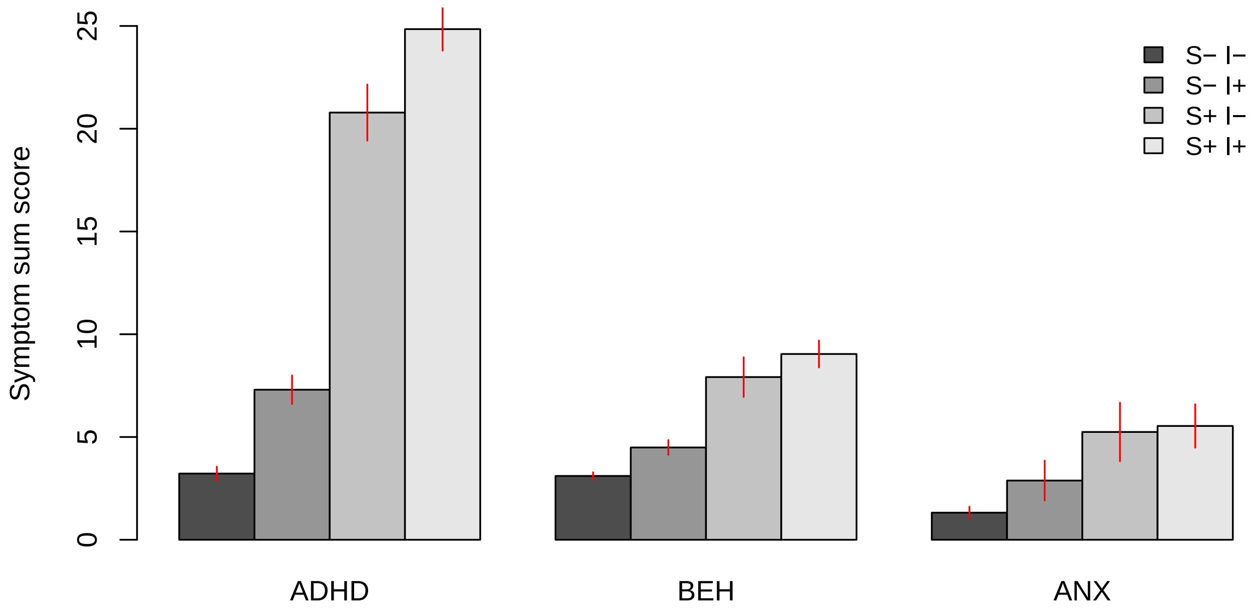


*Figure S2.* Symptoms sum scores for different diagnostic groups. "S− I−" = below symptom threshold, no impairment,"S− I+" = below symptom threshold, with impairment ,"S+ I−" = above symptom threshold, no impairment ,"S+ I+" = above symptom threshold, with impairment. As children are more clearly grouped by symptom load than by impingement, it is reasonable that symptom criteria play a greater role for classification. ADHD = Attention Deficit Hyperactivity Disorder, BEH = Behaviour problems, ANX = Anxiety problems.

**Calculation of scores for instrument sub-scales.** Scores for the NEPSY and Stanford Binet were calculated following standard procedures described in the manuals.

Scores for sub-scales of questionnaires were obtained by estimating latent Rasch models [56] with the edstan packages [57]. We used this approach as opposed to simply summing up scores and transforming them based on a norm-table, because Rasch models take better into account differences in item-difficulty and -informativeness. Moreover, norm-samples for many of the employed test are not from Europe or Norway, making the validity of normed scores from these tests for the ADHD Study questionable. Further, the explicit assumptions of an underlying

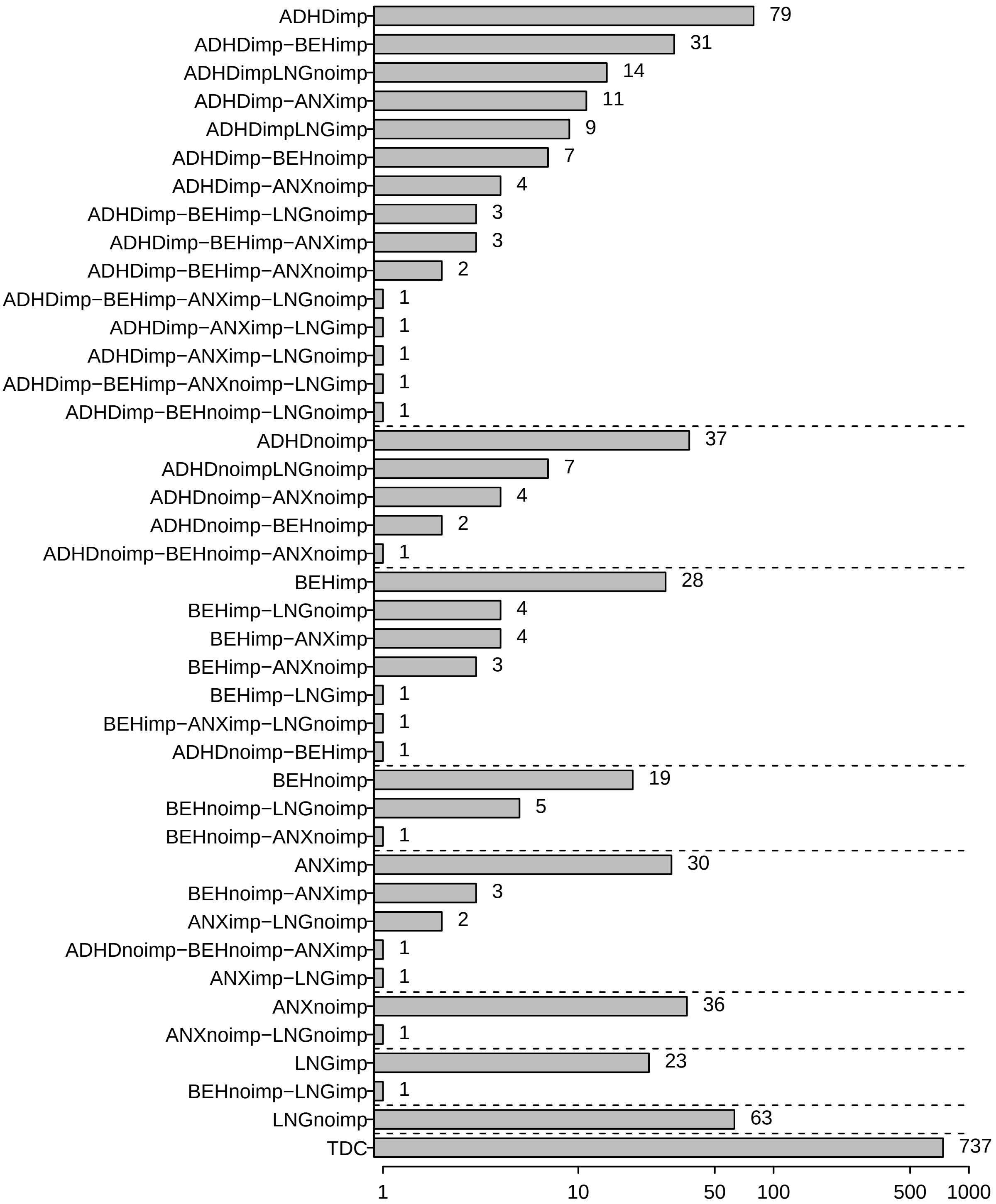


*Figure S3*. Combinations of mental health problems observed in the study. ADHD = Attention Deficit Hyperactivity Disorder, BEH = Behaviour problems, ANX = Anxiety problems, . . . imp = with impairment, . . . noimp = without impairment. Dotted horizontal lines delineate diagnostics groups used in the article.

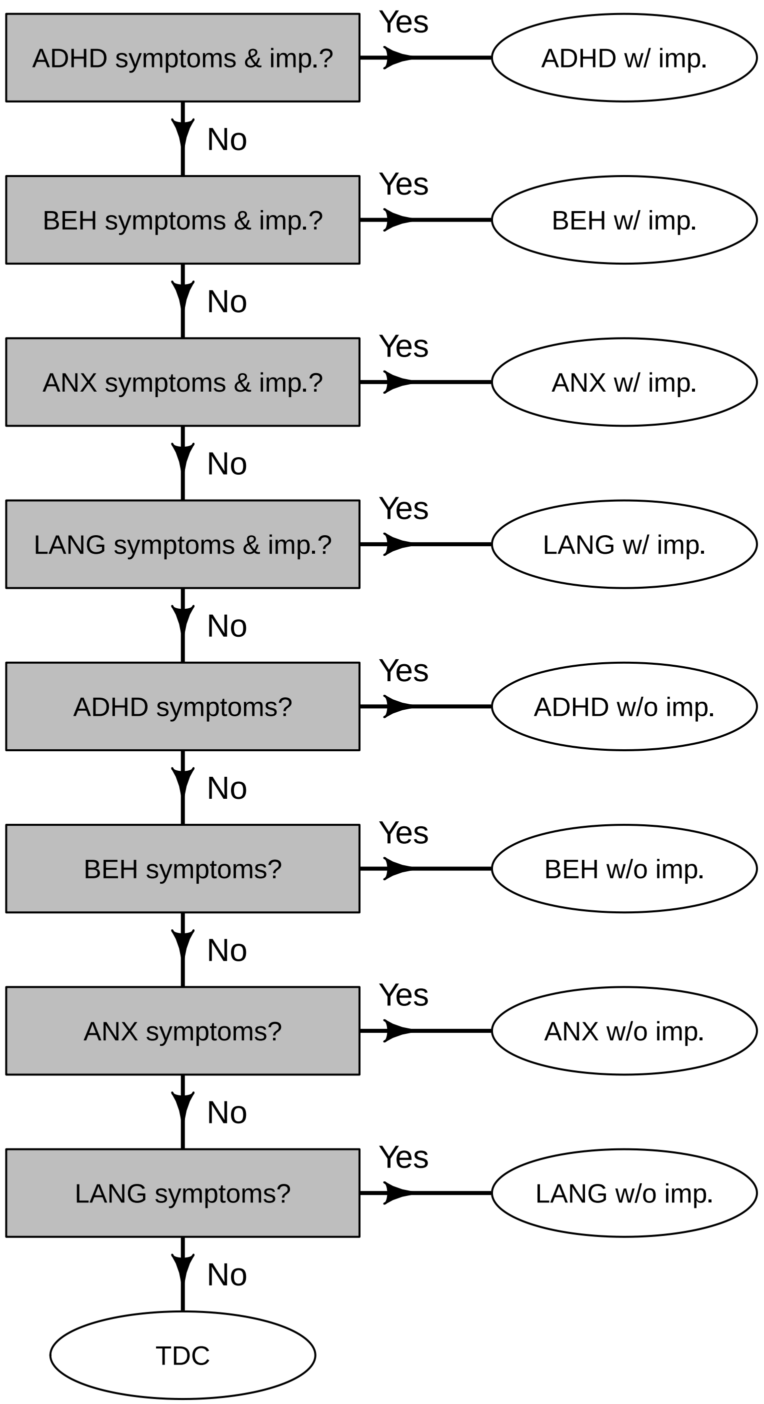


*Figure S4*. Decision tree for diagnostic classifications. For any mental health problems we first checked if children fulfilled all symptom and impairment criteria. Next we checked if children fulfilled symptom criteria. Children who did not fulfill any symptom criterion were classified as typically developing controls (TDC). ADHD = Attention Deficit Hyperactivity Disorder, BEH = Behaviour problems, ANX = Anxiety problems.

normally distributed trait that determines item responses particularly lends itself to the estimation of approximately normally distributed scores for all scales. This in turn facilitates all further analyses. We assigned items to sub-scales as described in the respective manuals, and then estimated a Rasch model for each sub-scale (e.g. five Rasch models for the 5 BRIEF sub-scales).

**Testing the RDoC domain structure.** We used factor-analytic methods to identify a smaller number of functional dimensions from altogether 33 scales, comprised of sub-scale scores from questionnaires and tests. A first analysis used confirmatory factor analyses (CFA) to test if a CFA model with theoretically guided assignment of the scales to the RDoC dimensions captures the co-variation between test scores. We tested CFAs that used either

1. all available sub-scales (*Positive Valence System*: CDT, CBQ high intensity pleasure, CBQ low intensity pleasure; *Negative valence System*: CBQ anxiety, CBQ fear, CBQ sadness, EAS emotional, CBQ soothing, CBQ perceptual sensitivity, CBQ discomfort, BRIEF emotional control, *Cognitive system*: BNT, BRIEF inhibition, BRIEF planning and organizing, BRIEF shift, BRIEF working memory, CDI, NEPSY inhibition, NEPSY language, NYVABU NYVSPD, Stanford Binet non-verbal IQ, Stanford Binet non-verbal working memory, Stanford Binet verbal IQ, Stanford Binet verbal fluid reasoning, Stanford Binet visual working memory, CBQ inhibitory control, CBQ attentional focusing, *Social Support system*: EAS shyness, EAS sociability, CBQ shyness; *Arousal and Regulatory Systems*: CBQ AL, EAS activity, CBQ impulsivity.

2. only sub-scales used for the final ESEM model (details below)

3. only sub-scales used for the final ESEM model which did not load on the introversion factor (see below)

4. as 3., and in addition sub-scales for the cognitive system were split into factor for

Table S1

*Fit indices for RDoC CFAs. The third model builds on the second and the fourth on the third.*

| Model Description | CFI | TLI | RMSEA | ... 90%CI LB | ... 90%CI UB |
|---|---|---|---|---|---|
| all subscales | 0.454 | 0.408 | 0.081 | 0.079 | 0.084 |
| final ESEM sub-scales | 0.759 | 0.716 | 0.070 | 0.067 | 0.074 |
| ... + no introv. sub-scales | 0.537 | 0.468 | 0.096 | 0.093 | 0.100 |
| ... + 2 CS factors | 0.522 | 0.463 | 0.090 | 0.087 | 0.092 |

questionnaire based and one for test based sub-scales.

The fit-indices Root Mean Square Error of Approximation (RMSEA) and Comparative Fit Index (CFI) shown in Table S1 indicate that these CFA models did not fit the data well. Hence we used exploratory structural equation modeling [ESEM, 58], as implemented in the software Mplus, to identify functional domains in our data.

**Identification of functional domains through exploratory structural equation modeling (ESEM).** We used an ESEM analysis instead of a classical exploratory factor analysis (EFA) because it allows identification of correlated factors and extraction of factor scores in Mplus. Most sub-scales scores were treated as normally distributed, because we calculated standardized scores (e.g. for the Stanford Binet tests), or because Rasch models assume and typically produce (approximately) normally distributed person-scores. The few sub-scale score that were clearly not normally distributed (CDT, NEPSY visual attention), were categorized into 8 ordered scores and analyzed as ordered categorical data.

To deal with missing data, ESEM models were estimated with the Full Information Maximum Likelihood Method.

We used a 2-step approach to identify a set of functional domains. First, we fitted ESEM models with two to ten latent factors (dimensions/domains) to all sub-scales and chose the ESEM model with the smallest number of factors that had an RMSEA below 0.05 and CFI above 0.95 as the best model. Based on this model, we identified non-valid sub-scales as those that had a low $r^2$ value ($r^2 < 0.2$). The reasoning here is that that sub-scales with a very low $r^2$ value are either unreliable, or measure something that is unrelated to all other sub-scales. Using only the valid sub-scales we performed a second ESEM analysis. Based on the results of the second ESEM we identified ambiguous sub-scales as those with high cross-loadings $\left(\frac{|highest\ loading|}{|2^{nd}\ highest\ loading|} < 1.25\right)$. Next we again fitted ESEM models with two to ten factors and identified the best model among these. Lastly, we investigated a possible simplification of the factor structure by testing CFA models in which low cross-loadings of scales were constrained to be zero.

**Comparison of functioning in preschoolers with and without ADHD.** We estimated a hierarchical regression model with the R package brms [59]. The basic regression model adjusts for maternal education and age, as well as for parity. The hierarchical parts of the model capture repeated measurement of individuals and model random effects for subsets of the data defined by by mental health problem, gender, and functional domain. The model also simultaneously imputed missing covariates. Specifically, the model was initially specified as follows:

effect_model = bf(value ~ 1 + mo(mEdu) + mo(parity) + Age + (1 | Domain:Gender:MHP) + (1|ID))

imputation_model = bf(mEdu ~ Age + poly(as.numeric(parity),2) + MHP, family = “cumulative”)

joint_model = effect_model + imputation_model + set_rescor(FALSE)

fit = brm(joint_model, data = factor_scores_long, family = “gaussian,” iter = 2000)

The model was fit with brms’ default weakly informative priors. In particular, fixed effects and variance parameters have a student-t prior with mean zero, standard deviation 3, and 10 degrees of freedom. These priors lead to a, compared to uniform priors, weak shrinkage of parameter estimates towards zero. Due to the large sample size of the ADHD Study (~1200 participants), priors have a negligible effect on the results.

To verify successful convergence of the Bayesian estimation, we checked potential scale reduction parameters ($\hat{R}$) and divergent iterations. $\hat{R}$ for all random parameters were below 1.1 and the chains had no divergent iterations.

## Self selection into the Study

To assess self selection into the ADHD Study, we performed Bayesian multiple regression analyses to predict participation based on mothers’ age and education (with the levels elementary school or less, high school, bachelor, or master degree), parity (0, 1, 2, 3 or more), and the child’s sex and ADHD sum score in the MoBa Q6. For control participants we also used participation in the Q6 as a predictor. For this analysis, missing data were imputed during estimation of regression weights in a custom Stan [60] program.

The results revealed some self selection in the ADHD Study. In the ADHD case group the chance to participate increased for each level of education, odds ratio (OR (95%) Highest Density Interval) 1.06 (1.04, 1.08), and increased substantially with each doubling of the ADHD sum score, OR 1.37 (1.23, 1.53). In the control group, the chance to participate was much lower for participants who had not returned the Q6, OR (95% Highest Density Interval, HDI) 0.68 (0.63, 0.72), ORs were 0.99 (0.96,

1.03) and 1.04 (0.99, 1.12) for education and ADHD scum score, respectively.

## Supplementary results: Factor loadings and correlations

Initial CFAs, for which sub-scales were on theoretical grounds assigned to RDoC domains, did not describe the data sufficiently well (RMSEA = 0.09 (0.087,0.092), CFI = 0.52, TLI = 0.46, see also Table S1 ). The first ESEM analysis including all 34 sub-scales showed that following scales either had an $r^2 < 0.2$ or high cross-loadings on a second factor and were thus removed from the analysis: BRIEF-Shift, CBQ-Discomfort, -Fear, and -Inhibitory control, NEPSY language test, Stanford Binet's test for nonverbal IQ and verbal fluency. The final ESEM analysis with the remaining 26 sub-scales showed that a 7-factor model was the simplest model that could adequately describe the data (RMSEA = 0.03 (0.028, 0.037), CFI = 0.96). Follow up CFA analyses that constrained small cross loadings to 0 resulted in unsatisfactory RMSEA and CFI statistics. We hence retained the final ESEM model as the best model of functional domains for preschoolers in our sample.

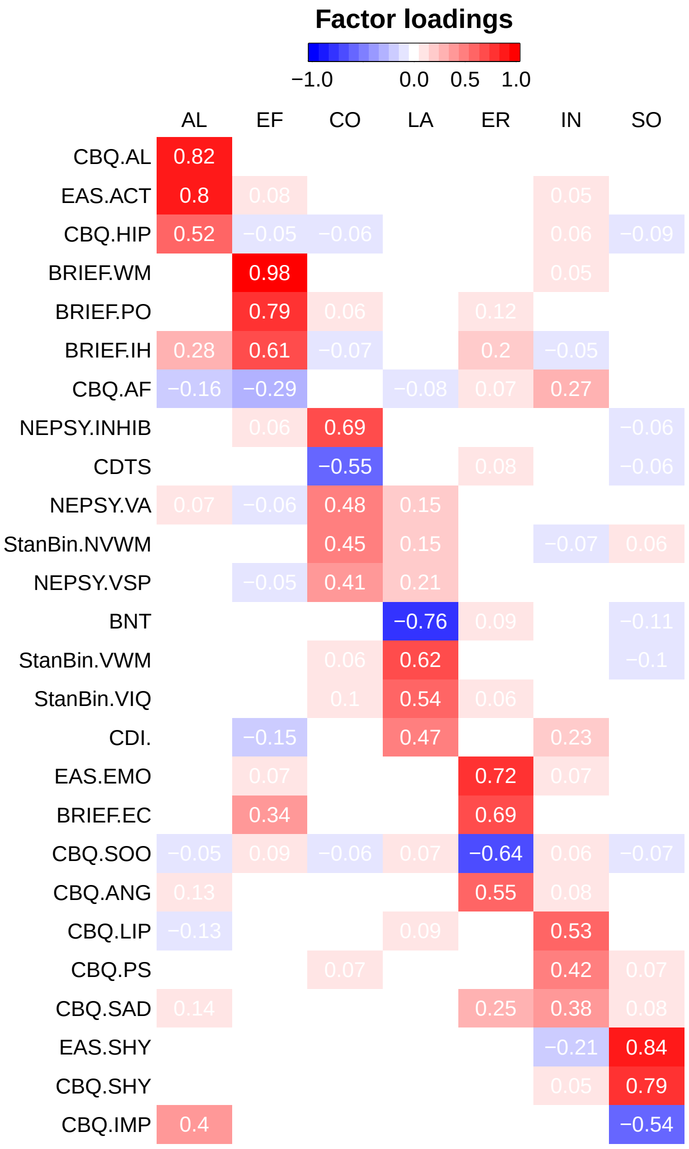


*Figure S5*. Factor loadings for the final ESEM model. AL = Activity level and regulation, EF = Executive functions, CO = cognition, LA = Language, ER = Emotion regulation, IN = Introversion, SO = Socialbility. See methods section for abbreviations of instruments and subscales.

Table S2

*x- and y- standardized factor loadings with standard errors*

| | AL | EF | CO | LA | ER | IN | SO |
|---|---|---|---|---|---|---|---|
| BNT | -0.04 (0.03) | -0.03 (0.03) | 0.03 (0.03) | -0.76 (0.04) | 0.09 (0.04) | 0.01 (0.03) | -0.11 (0.05) |
| BRIEF.EC | -0.04 (0.02) | 0.34 (0.04) | -0.00 (0.02) | 0.03 (0.02) | 0.69 (0.03) | -0.03 (0.01) | 0.01 (0.01) |
| BRIEF.IH | 0.28 (0.02) | 0.61 (0.02) | -0.07 (0.02) | 0.02 (0.02) | 0.20 (0.03) | -0.05 (0.02) | -0.01 (0.02) |
| BRIEF.PO | -0.00 (0.01) | 0.79 (0.02) | 0.06 (0.02) | -0.02 (0.02) | 0.12 (0.03) | -0.01 (0.02) | -0.02 (0.02) |
| BRIEF.WM | 0.01 (0.01) | 0.98 (0.01) | 0.01 (0.01) | -0.04 (0.02) | -0.03 (0.01) | 0.05 (0.02) | 0.03 (0.01) |
| CBQ.AF | -0.16 (0.04) | -0.29 (0.03) | 0.02 (0.03) | -0.08 (0.04) | 0.07 (0.03) | 0.27 (0.04) | -0.00 (0.03) |
| CBQ.AL | 0.82 (0.02) | 0.01 (0.01) | 0.03 (0.02) | -0.04 (0.02) | 0.02 (0.02) | -0.03 (0.02) | 0.01 (0.02) |
| CBQ.ANG | 0.13 (0.03) | 0.03 (0.02) | -0.04 (0.03) | -0.02 (0.03) | 0.55 (0.03) | 0.08 (0.03) | -0.03 (0.03) |
| CBQ.HIP | 0.52 (0.03) | -0.05 (0.03) | -0.06 (0.04) | 0.02 (0.03) | 0.02 (0.03) | 0.06 (0.03) | -0.09 (0.03) |
| CBQ.IMP | 0.40 (0.03) | -0.01 (0.02) | -0.02 (0.03) | 0.04 (0.03) | 0.01 (0.02) | -0.04 (0.02) | -0.54 (0.02) |
| CBQ.LIP | -0.13 (0.04) | -0.01 (0.02) | -0.04 (0.03) | 0.09 (0.04) | -0.03 (0.03) | 0.53 (0.04) | -0.03 (0.02) |
| CBQ.PS | 0.03 (0.02) | -0.03 (0.03) | 0.07 (0.05) | 0.03 (0.03) | -0.02 (0.03) | 0.42 (0.04) | 0.07 (0.04) |
| CBQ.SAD | 0.14 (0.04) | 0.00 (0.02) | 0.00 (0.03) | -0.04 (0.03) | 0.25 (0.04) | 0.38 (0.04) | 0.08 (0.03) |
| CBQ.SHY | 0.03 (0.02) | 0.03 (0.02) | -0.01 (0.02) | 0.03 (0.02) | 0.01 (0.02) | 0.05 (0.03) | 0.79 (0.02) |
| CBQ.SOO | -0.05 (0.02) | 0.09 (0.03) | -0.06 (0.04) | 0.07 (0.03) | -0.64 (0.03) | 0.06 (0.03) | -0.07 (0.03) |
| CDI. | 0.01 (0.02) | -0.15 (0.04) | 0.01 (0.03) | 0.47 (0.03) | 0.01 (0.02) | 0.23 (0.04) | -0.02 (0.02) |
| CDTS | 0.01 (0.03) | -0.03 (0.03) | -0.55 (0.05) | 0.03 (0.04) | 0.08 (0.04) | 0.02 (0.03) | -0.06 (0.04) |

Table S2 continued

| | AL | EF | CO | LA | ER | IN | SO |
|---|---|---|---|---|---|---|---|
| EAS.ACT | 0.80 (0.02) | 0.08 (0.02) | 0.01 (0.02) | -0.01 (0.02) | -0.04 (0.02) | 0.05 (0.02) | -0.02 (0.02) |
| EAS.EMO | -0.04 (0.02) | 0.07 (0.03) | -0.02 (0.03) | 0.03 (0.02) | 0.72 (0.03) | 0.07 (0.03) | -0.01 (0.02) |
| EAS.SHY | -0.04 (0.02) | -0.03 (0.02) | 0.00 (0.02) | 0.00 (0.01) | 0.03 (0.02) | -0.21 (0.04) | 0.84 (0.03) |
| NEPSY.INHIB | -0.02 (0.03) | 0.06 (0.03) | 0.69 (0.04) | -0.02 (0.03) | 0.00 (0.02) | 0.04 (0.03) | -0.06 (0.03) |
| NEPSY.VA | 0.07 (0.04) | -0.07 (0.04) | 0.48 (0.05) | 0.15 (0.05) | 0.04 (0.04) | 0.00 (0.04) | 0.01 (0.03) |
| NEPSY.VSP | -0.03 (0.03) | -0.05 (0.03) | 0.41 (0.04) | 0.21 (0.05) | 0.04 (0.03) | 0.03 (0.04) | -0.03 (0.03) |
| StanBin.NVWM | 0.00 (0.03) | -0.02 (0.03) | 0.45 (0.05) | 0.15 (0.05) | -0.04 (0.03) | -0.07 (0.04) | 0.06 (0.03) |
| StanBin.VIQ | -0.04 (0.03) | -0.01 (0.03) | 0.10 (0.05) | 0.55 (0.04) | 0.06 (0.03) | 0.01 (0.03) | -0.01 (0.02) |
| StanBin.VWM | -0.03 (0.03) | 0.01 (0.03) | 0.06 (0.04) | 0.62 (0.04) | 0.01 (0.02) | -0.02 (0.03) | -0.10 (0.04) |

*Note.* Numbers are mean and confidence intervals. AL = Activity level and regulation, EF = Executive functions, CO = cognition, LA = Language, ER = Emotion regulation, IN = Introversion, SO = Socialbility. See methods section for abbreviations of instruments and subscales.

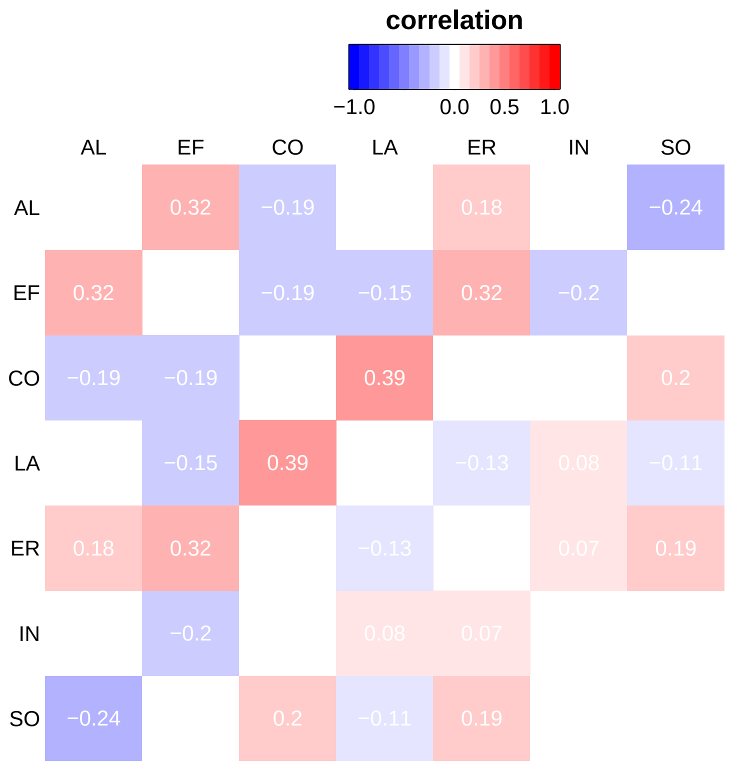


*Figure S6*. Factor correlations for the final ESEM model. AL = Activity level and regulation, EF = Executive functions, CO = cognition, LA = Language, ER = Emotion regulation, IN = Introversion, SO = Socialbility.

Table S3

*Factor correlations with confidence intervals*

| | EF | CO | LA | ER | IN | SO |
|---|---|---|---|---|---|---|
| AL | 0.33 (-0.31, 0.96) | 0.32 (-0.31, 0.96) | -0.15 ( 0.14, -0.45) | 0.03 (-0.03, 0.08) | -0.19 ( 0.19, -0.57) | -0.20 ( 0.19, -0.59) |
| EF | | 0.18 (-0.17, 0.53) | -0.13 ( 0.12, -0.37) | 0.19 (-0.18, 0.56) | -0.01 ( 0.01, -0.03) | 0.07 (-0.07, 0.20) |
| CO | | | -0.04 ( 0.04, -0.11) | -0.24 ( 0.23, -0.72) | -0.19 ( 0.18, -0.56) | -0.02 ( 0.02, -0.06) |
| LA | | | | -0.11 ( 0.10, -0.31) | 0.39 (-0.37, 1.15) | 0.08 (-0.08, 0.24) |
| ER | | | | | 0.20 (-0.20, 0.60) | 0.04 (-0.04, 0.13) |
| IN | | | | | | 0.03 (-0.03, 0.09) |

*Note.* Numbers are means and confidence intervals. AL = Activity level and regulation, EF = Executive functions, CO = cognition, LA = Language, ER = Emotion regulation, IN = Introversion, SO = Socialbility.

Table S4

*Average differences in functioning across domains between preschoolers with mental health problems and typically developing preschoolers*

| | with impairment | without impairment |
|---|---|---|
| ADHD | -0.55 (-0.62, -0.48; 0.91) | -0.28 (-0.40, -0.17; 0.00) |
| BEH | -0.33 (-0.45, -0.21; 0.01) | -0.29 (-0.44, -0.15; 0.00) |
| ANX | -0.20 (-0.33, -0.08; 0.00) | -0.17 (-0.29, -0.04; 0.00) |
| LNG | -0.52 (-0.66, -0.36; 0.60) | -0.30 (-0.41, -0.20; 0.00) |

*Note.* Standardized mean deviations (SMD). Values are mean (lower 90% HDI, upper 90% HDI; P(SMD<-0.5)). ADHD = preschoolers with AHDH problems, BEH = behavior problems, ANX = anxiety, LNG = language.

**Supplementary results: Group comparisons**

Table S5

*Differences in functioning between preschoolers with ADHD and typically developing preschoolers, stratified by gender or severity of mental health problem.*

| | V1 | with impairment | without impairment | boy | girl |
|---|---|---|---|---|---|
| AL | -0.92 (-1.07, -0.78; 1.00) | -0.97 (-1.14, -0.82; 1.00) | -0.74 (-0.99, -0.47; 0.97) | -0.80 (-1.01, -0.57; 1.00) | -0.92 (-1.14, -0.70; 1.00) |
| EF | -0.91 (-1.04, -0.77; 1.00) | -1.02 (-1.17, -0.87; 1.00) | -0.58 (-0.82, -0.33; 0.74) | -0.73 (-0.93, -0.51; 0.98) | -0.86 (-1.07, -0.65; 1.00) |
| CO | -0.51 (-0.65, -0.37; 0.58) | -0.55 (-0.70, -0.40; 0.73) | -0.40 (-0.66, -0.17; 0.22) | -0.46 (-0.67, -0.25; 0.37) | -0.49 (-0.70, -0.26; 0.45) |
| LA | -0.43 (-0.55, -0.28; 0.16) | -0.53 (-0.68, -0.38; 0.67) | -0.09 (-0.34, 0.15; 0.00) | -0.28 (-0.49, -0.07; 0.02) | -0.34 (-0.55, -0.12; 0.08) |
| ER | -0.62 (-0.75, -0.47; 0.95) | -0.71 (-0.86, -0.55; 0.99) | -0.31 (-0.56, -0.06; 0.07) | -0.44 (-0.65, -0.23; 0.30) | -0.58 (-0.80, -0.36; 0.75) |
| IN | -0.11 (-0.24, 0.04; 0.00) | -0.18 (-0.32, -0.01; 0.00) | 0.12 (-0.13, 0.36; 0.00) | -0.12 (-0.32, 0.11; 0.00) | 0.06 (-0.17, 0.27; 0.00) |
| SO | 0.09 (-0.07, 0.22; 0.00) | 0.11 (-0.05, 0.27; 0.00) | 0.02 (-0.21, 0.26; 0.00) | -0.14 (-0.35, 0.08; 0.00) | 0.26 ( 0.05, 0.49; 0.00) |

*Note.* AL = Activity level and regulation, EF = Executive functions, CO = cognition, LA = Language, ER = Emotion regulation, IN = Introversion, SO = Socialbility. Values are standardized mean deviations (lower and upper 90% credible interval; P(SMD<-0.5))

**Comparison of ADHD and ABC-Study samples**

The sample of the ADHD study does not include children with ASD, because these children were recruited into the ABC Study, a sister study about ASD. The following plots show data for tests and questionnaires that were available for both study (for the ABC Study, only around 50 of the full samples has data from the CBQ and EAS).

Children in the ABC study were classified into groups following the same algorithm described above. For the following analysis, each group with a mental health problems includes children with clinical and sub-clinical[2] classification.

The following figures show largely consistent results for the comparison of children with an without ADHD. One exception are children with language problems: Children with language problems in the ABC Study have greater language related problems than children with language problems in the ADHD study. This is likely explained by the fact that the ABC's studies attempt to recruit children with ASD also resulted in recruitment of children with sever language problems.

[2] i.e. they full symptom-criteria but have only limited impairments, or they have clear impairments despite not fulfilling symptom criteria

Table S6

*Differences in functioning between preschoolers with ADHD and other mental health problems.*

| Comparison Group | Domain | ADHD w/ impairm. | ADHD w/o impairm. |
|---|---|---|---|
| ANX w/ impairm. | AL | -1.29 (-1.57, -0.98; 1.00) | -1.06 (-1.43, -0.71; 1.00) |
| | EF | -0.84 (-1.13, -0.52; 0.98) | -0.39 (-0.75, -0.04; 0.28) |
| | CO | -0.72 (-1.02, -0.42; 0.93) | -0.58 (-0.92, -0.20; 0.67) |
| | LA | -0.30 (-0.61, 0.01; 0.11) | 0.14 (-0.20, 0.51; 0.00) |
| | ER | -0.17 (-0.46, 0.13; 0.01) | 0.23 (-0.13, 0.57; 0.00) |
| | IN | -0.24 (-0.57, 0.06; 0.05) | 0.05 (-0.32, 0.39; 0.00) |
| | SO | 1.12 ( 0.82, 1.42; 0.00) | 1.03 ( 0.66, 1.36; 0.00) |
| BEH w/ impairm. | AL | -0.73 (-1.02, -0.44; 0.94) | -0.50 (-0.85, -0.15; 0.49) |
| | EF | -0.56 (-0.86, -0.27; 0.65) | -0.12 (-0.45, 0.22; 0.01) |
| | CO | -0.44 (-0.72, -0.15; 0.34) | -0.29 (-0.65, 0.04; 0.12) |
| | LA | -0.15 (-0.44, 0.14; 0.01) | 0.30 (-0.04, 0.65; 0.00) |
| | ER | 0.18 (-0.11, 0.47; 0.00) | 0.58 ( 0.23, 0.90; 0.00) |
| | IN | -0.08 (-0.36, 0.21; 0.00) | 0.21 (-0.15, 0.55; 0.00) |
| | SO | 0.26 (-0.03, 0.55; 0.00) | 0.17 (-0.16, 0.52; 0.00) |
| LNG w/ impairm. | AL | -1.11 (-1.49, -0.76; 1.00) | -0.88 (-1.28, -0.48; 0.97) |
| | EF | -0.42 (-0.82, -0.08; 0.34) | 0.02 (-0.42, 0.41; 0.01) |
| | CO | 0.21 (-0.17, 0.56; 0.00) | 0.36 (-0.04, 0.77; 0.00) |
| | LA | 1.20 ( 0.83, 1.59; 0.00) | 1.65 ( 1.25, 2.11; 0.00) |
| | ER | -0.52 (-0.87, -0.14; 0.54) | -0.12 (-0.52, 0.28; 0.03) |
| | IN | 0.32 (-0.05, 0.66; 0.00) | 0.61 ( 0.21, 1.02; 0.00) |
| | SO | 0.10 (-0.24, 0.45; 0.00) | 0.01 (-0.38, 0.41; 0.01) |

*Note.* AL = Activity level and regulation, EF = Executive functions, CO = cognition, LA = Language, ER = Emotion regulation, IN = Introversion, SO = Socialbility. Values are standardized mean deviations (lower and upper 90% credible interval; P(SMD<-0.5))

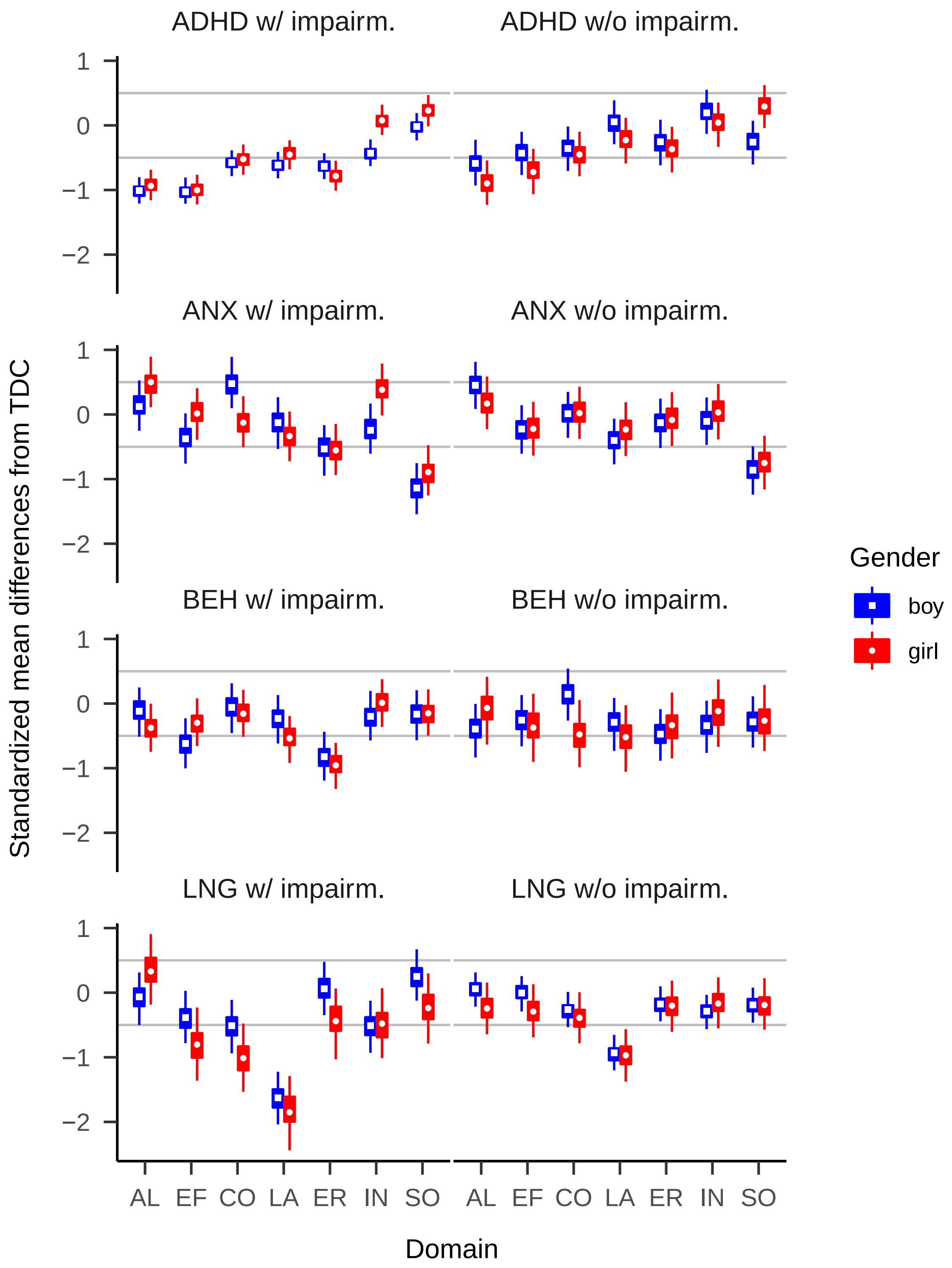


*Figure S7.* Comparison of children with mental health problems against typically developing controls, stratified by mental health problem type and gender. AL = Activity level and regulation, EF = Executive functions, CO = cognition, LA = Language, ER = Emotion regulation, IN = Introversion, SO = Socialbility. ADHD = preschoolers with AHDH problems, BEH = behavior problems, ANX = anxiety, LNG = language.

Table S7

*Differences in functioning between preschoolers with ADHD and other mental health problems by domain and severity.*

| Comparison Group | Domain | w/ impairm. | w/o impairm. |
|---|---|---|---|
| ALL | AL | -1.04 (-1.47, -0.57; 0.98) | -0.74 (-1.25, -0.29; 0.78) |
| | EF | -0.61 (-1, -0.22; 0.66) | -0.35 (-0.66, -0.01; 0.22) |
| | CO | -0.32 (-0.89, 0.4; 0.42) | -0.24 (-0.63, 0.13; 0.14) |
| | LA | 0.25 (-0.48, 1.41; 0.04) | 0.47 (-0.01, 1.04; 0) |
| | ER | -0.17 (-0.71, 0.34; 0.18) | -0.08 (-0.43, 0.31; 0.02) |
| | IN | 0 (-0.41, 0.51; 0.02) | 0.28 (-0.07, 0.62; 0) |
| | SO | 0.49 (-0.09, 1.28; 0) | 0.44 (-0.02, 1.01; 0) |
| BEH | AL | -0.73 (-0.97, -0.48; 0.94) | -0.51 (-0.84, -0.19; 0.52) |
| | EF | -0.56 (-0.8, -0.31; 0.65) | -0.26 (-0.61, 0.08; 0.13) |
| | CO | -0.44 (-0.68, -0.19; 0.34) | -0.24 (-0.57, 0.11; 0.09) |
| | LA | -0.15 (-0.39, 0.09; 0.01) | 0.32 (-0.02, 0.66; 0) |
| | ER | 0.18 (-0.06, 0.43; 0) | 0.09 (-0.24, 0.43; 0) |
| | IN | -0.08 (-0.33, 0.16; 0) | 0.34 (0.01, 0.68; 0) |
| | SO | 0.26 (0.02, 0.51; 0) | 0.29 (-0.05, 0.62; 0) |
| ANX | AL | -1.29 (-1.54, -1.03; 1) | -1.05 (-1.35, -0.76; 1) |
| | EF | -0.84 (-1.09, -0.58; 0.98) | -0.35 (-0.65, -0.05; 0.21) |
| | CO | -0.72 (-0.97, -0.47; 0.93) | -0.42 (-0.72, -0.12; 0.33) |
| | LA | -0.3 (-0.57, -0.04; 0.11) | 0.23 (-0.06, 0.52; 0) |
| | ER | -0.17 (-0.42, 0.08; 0.01) | -0.2 (-0.52, 0.1; 0.06) |
| | IN | -0.24 (-0.51, 0.02; 0.05) | 0.15 (-0.15, 0.46; 0) |
| | SO | 1.12 (0.87, 1.38; 0) | 0.82 (0.52, 1.13; 0) |
| LNG | AL | -1.11 (-1.42, -0.81; 1) | -0.65 (-0.94, -0.36; 0.81) |
| | EF | -0.42 (-0.73, -0.12; 0.34) | -0.43 (-0.7, -0.15; 0.34) |
| | CO | 0.21 (-0.08, 0.52; 0) | -0.08 (-0.36, 0.21; 0.01) |
| | LA | 1.2 (0.89, 1.53; 0) | 0.86 (0.58, 1.14; 0) |
| | ER | -0.52 (-0.82, -0.21; 0.54) | -0.12 (-0.4, 0.16; 0.01) |
| | IN | 0.32 (0.01, 0.62; 0) | 0.35 (0.07, 0.62; 0) |
| | SO | 0.1 (-0.19, 0.39; 0) | 0.21 (-0.06, 0.47; 0) |

*Note.* Values are standardized mean deviations (lower and upper 90% credible interval; P(SMD<-0.5)). ADHD = preschoolers with AHDH problems, BEH = behavior problems, ANX = anxiety, LNG = language. AL = Activity level and regulation, EF = Executive functions, CO = cognition, LA = Language, ER = Emotion regulation, IN = Introversion, SO = Socialbility.

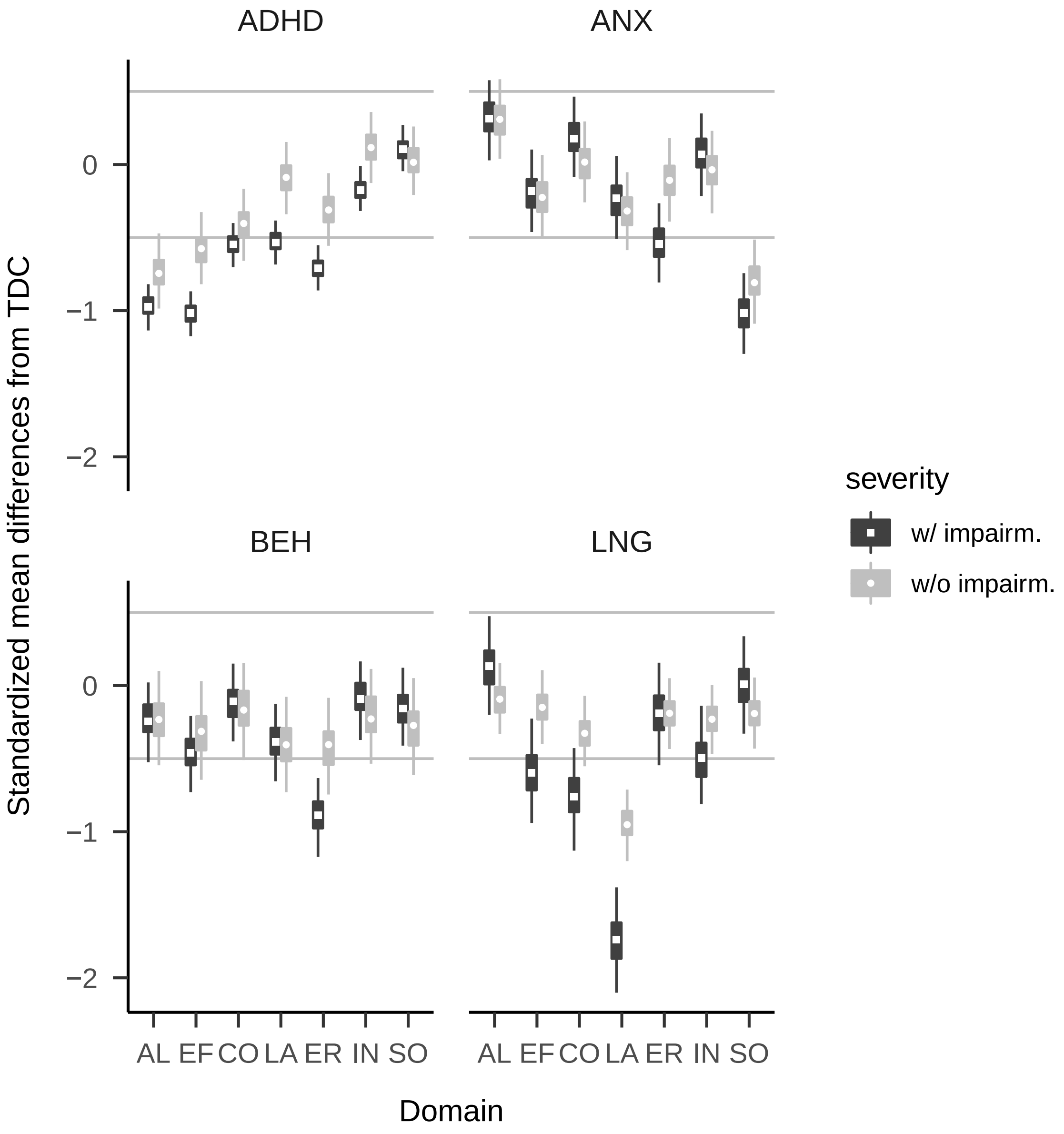


*Figure S8*. Comparison of children with mental health problems against typically developing controls, stratified by mental health problem type and problem severity (clinical and sub-clinical). AL = Activity level and regulation, EF = Executive functions, CO = cognition, LA = Language, ER = Emotion regulation, IN = Introversion, SO = Socialbility. ADHD = preschoolers with AHDH problems, BEH = behavior problems, ANX = anxiety, LNG = language.

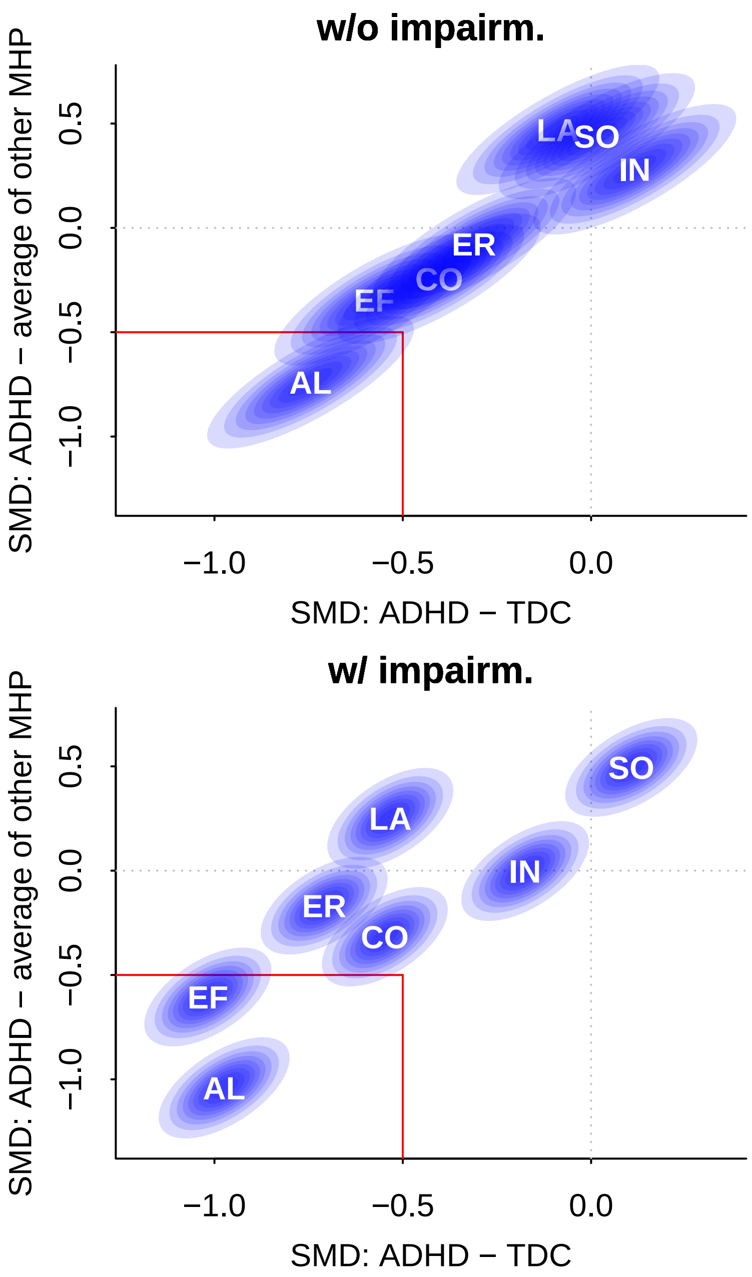


*Figure S9*. Comparison of preschoolers with ADHD with typically developing preschoolers and preschoolers with other *clinical* mental health problems, stratified by severity of impairment. Values below 0 indicate that the comparison group has fewer problems than preschoolers with ADHD. Ellipses cover 90% HDIs. Labels in ellipses indicate functional domains. Domains below the diagonal line are those where the sum of the SMD differences to TDCs and other mental health problems is 1. Domains in the lower left rectangle are those where the SMD differences to both TDCs and other mental health problems is at least 0.5.

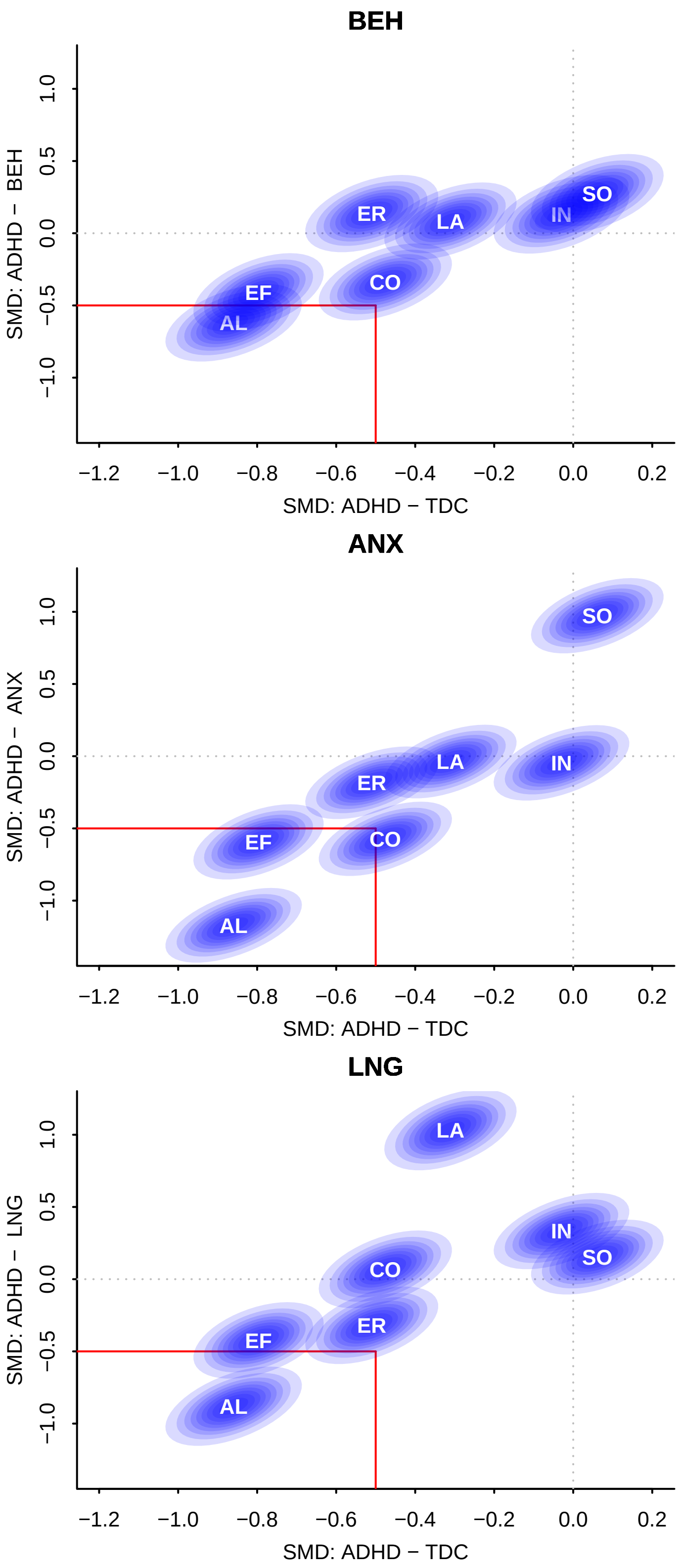


*Figure S10*. Comparison of preschoolers with ADHD with typically developing preschoolers and preschoolers with other *clinical* mental health problems.

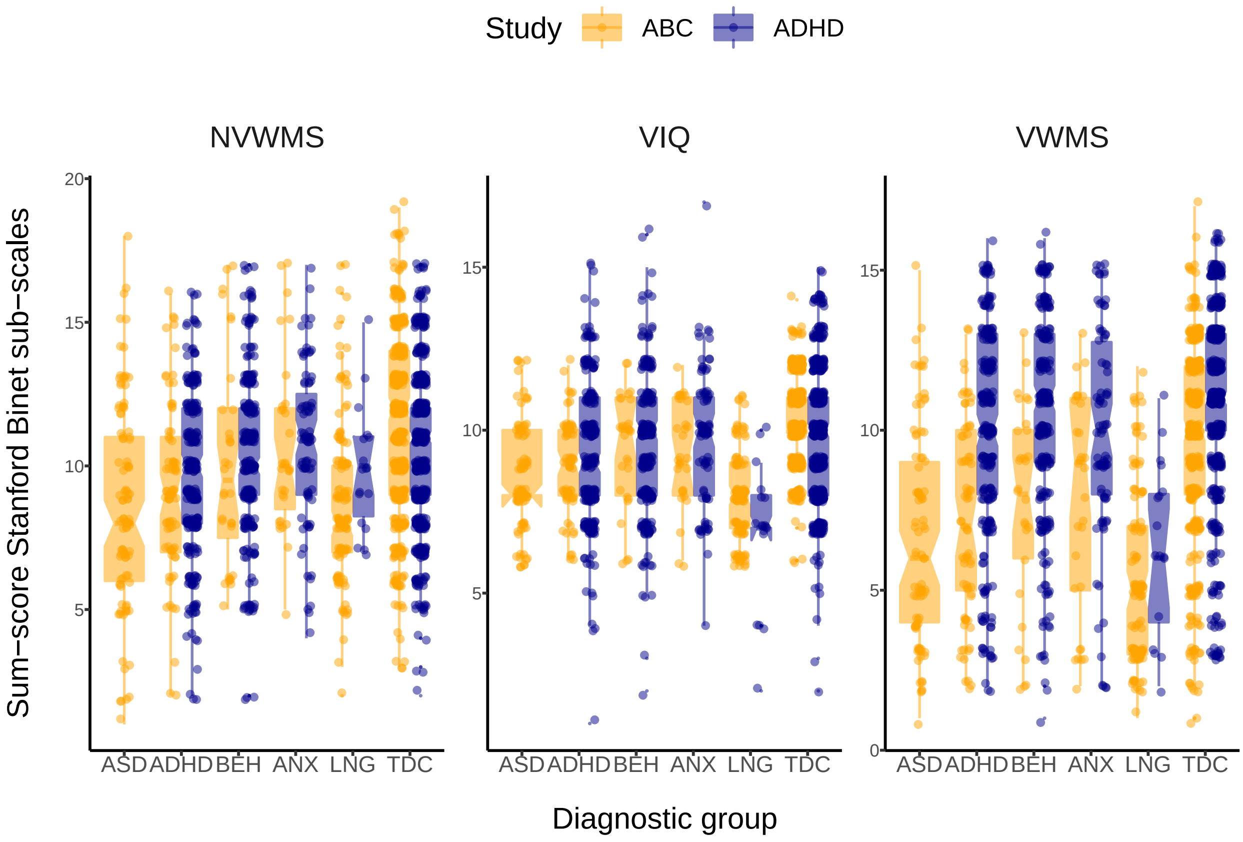


*Figure S11.* Stanford Binet Test results in the ADHD and ABC studies. Individual data points (dots) and boxplots with median, 25% and 75% quatiles. Notches, which are calculated as 1.58 * interquartile range divided by the squareroot of n, approximate 95% confidence intervals.

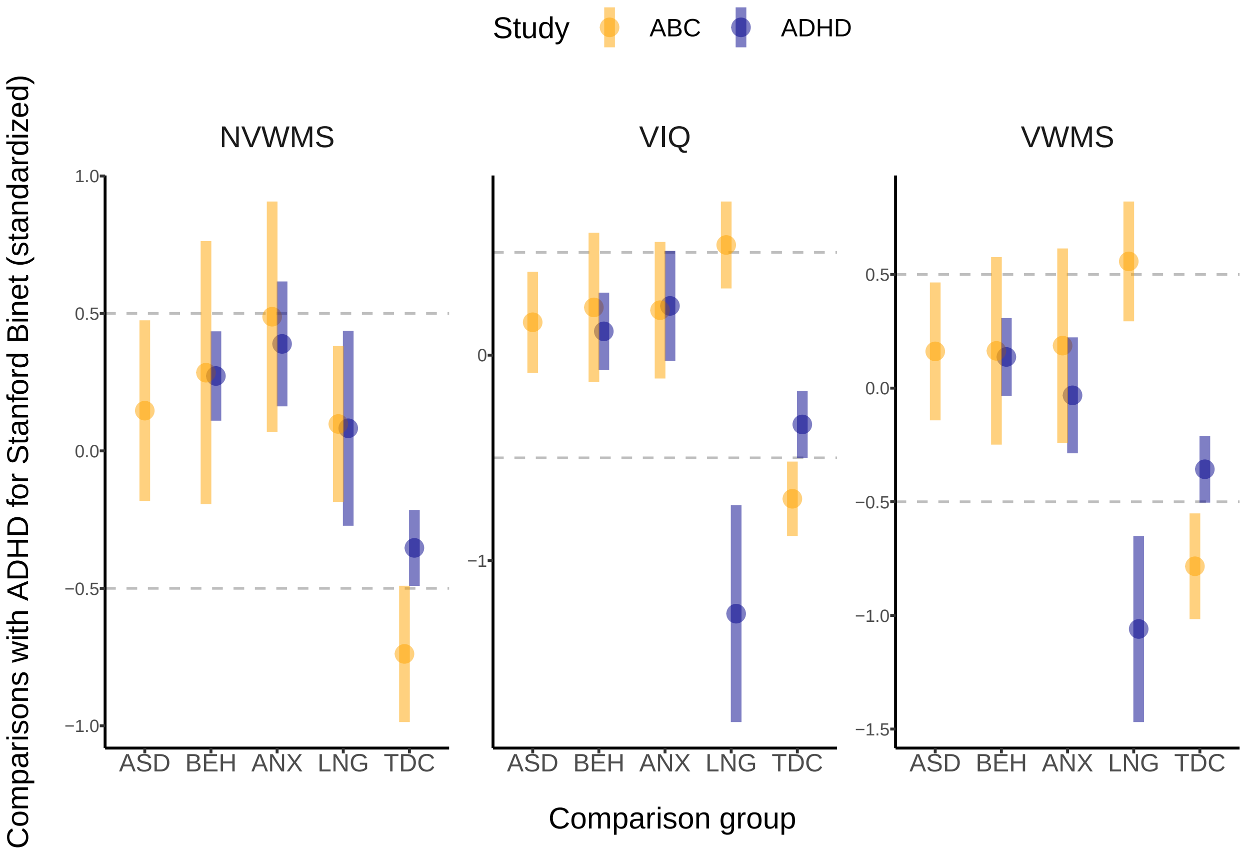


*Figure S12*. Comparison of children from ADHD and ABC Studies across Stanford Binet Test results. All differences are based on scaled scores, whereby scaling was implemented by dividing the raw scores by the standardeviation of all scores (across sub-groups and studies).

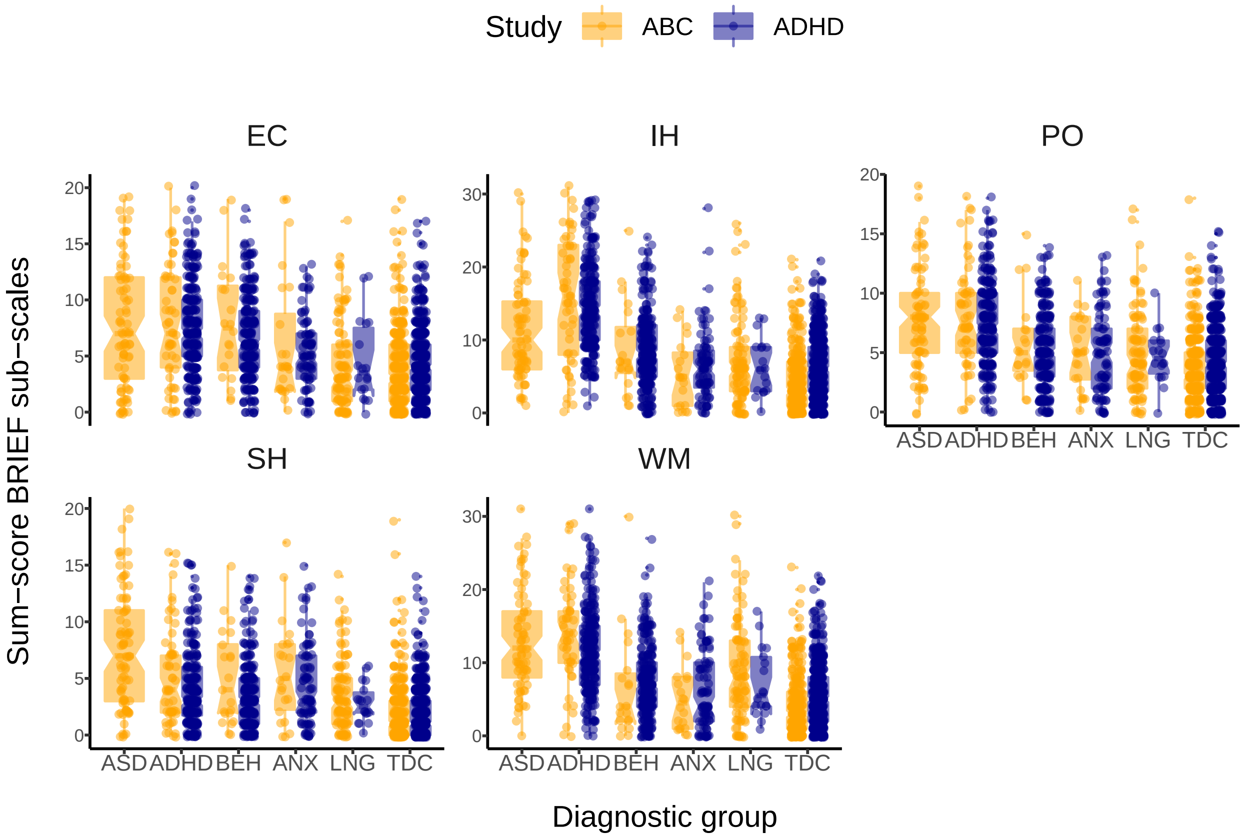


*Figure S13*. BRIEF results in the ADHD and ABC studies, stratified by group.

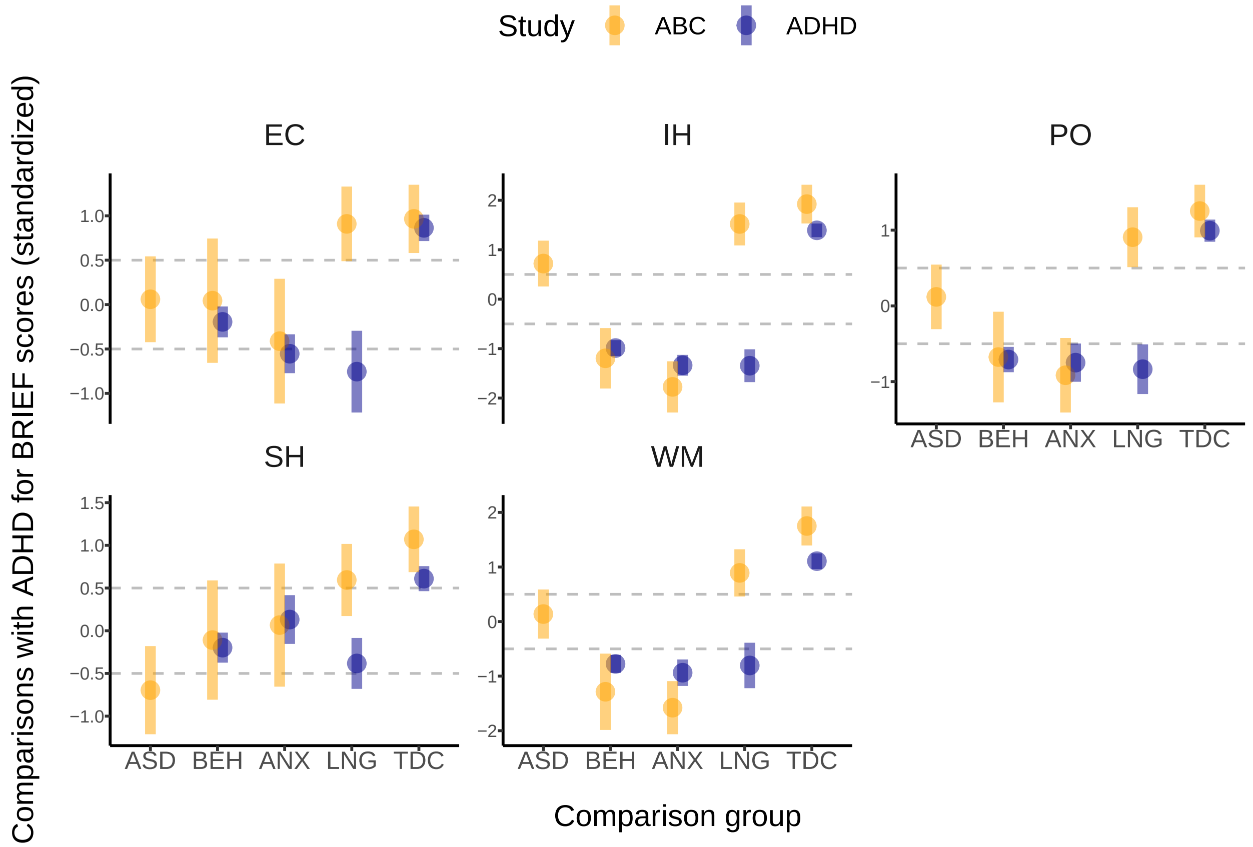


*Figure S14.* Comparison of children from ADHD and ABC Studies across BRIEF results. All differences are based on scaled scores, whereby scaling was implemented by deviding the raw scores by the standardeviation of all scores (across sub-groups and studies).

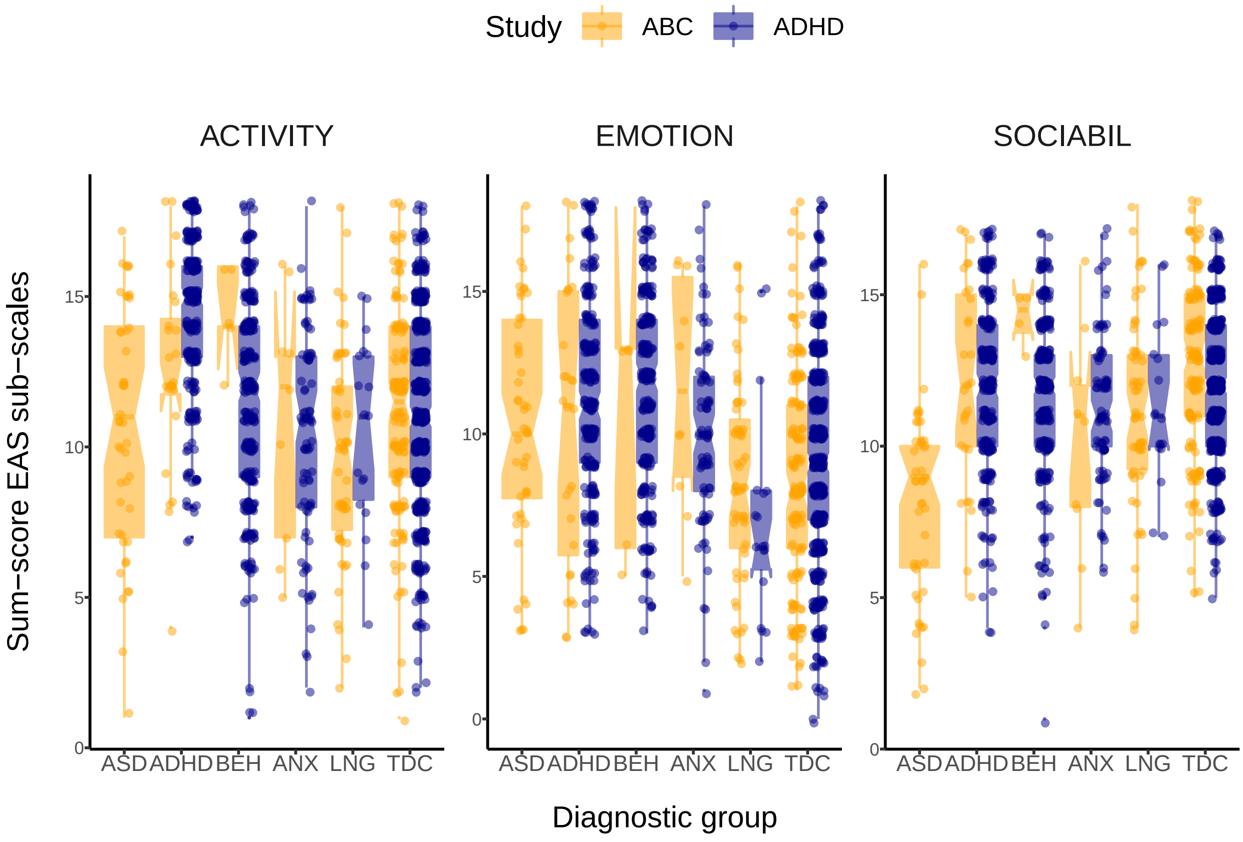


*Figure S15*. EAS results in the ADHD and ABC studies, stratified by group.

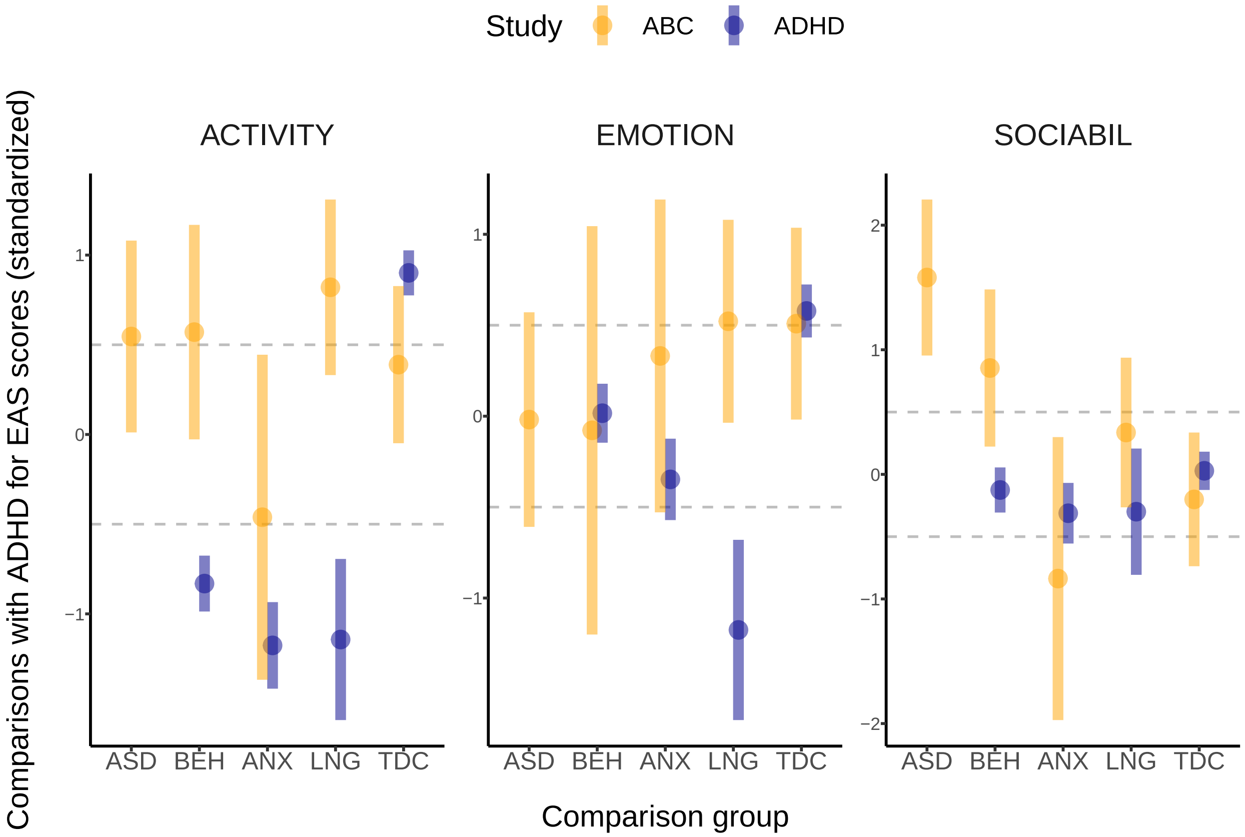


*Figure S16*. Comparison of children from ADHD and ABC Studies across EAS results. All differences are based on scaled scores, whereby scaling was implemented by deviding the raw scores by the standardeviation of all scores (across sub-groups and studies).

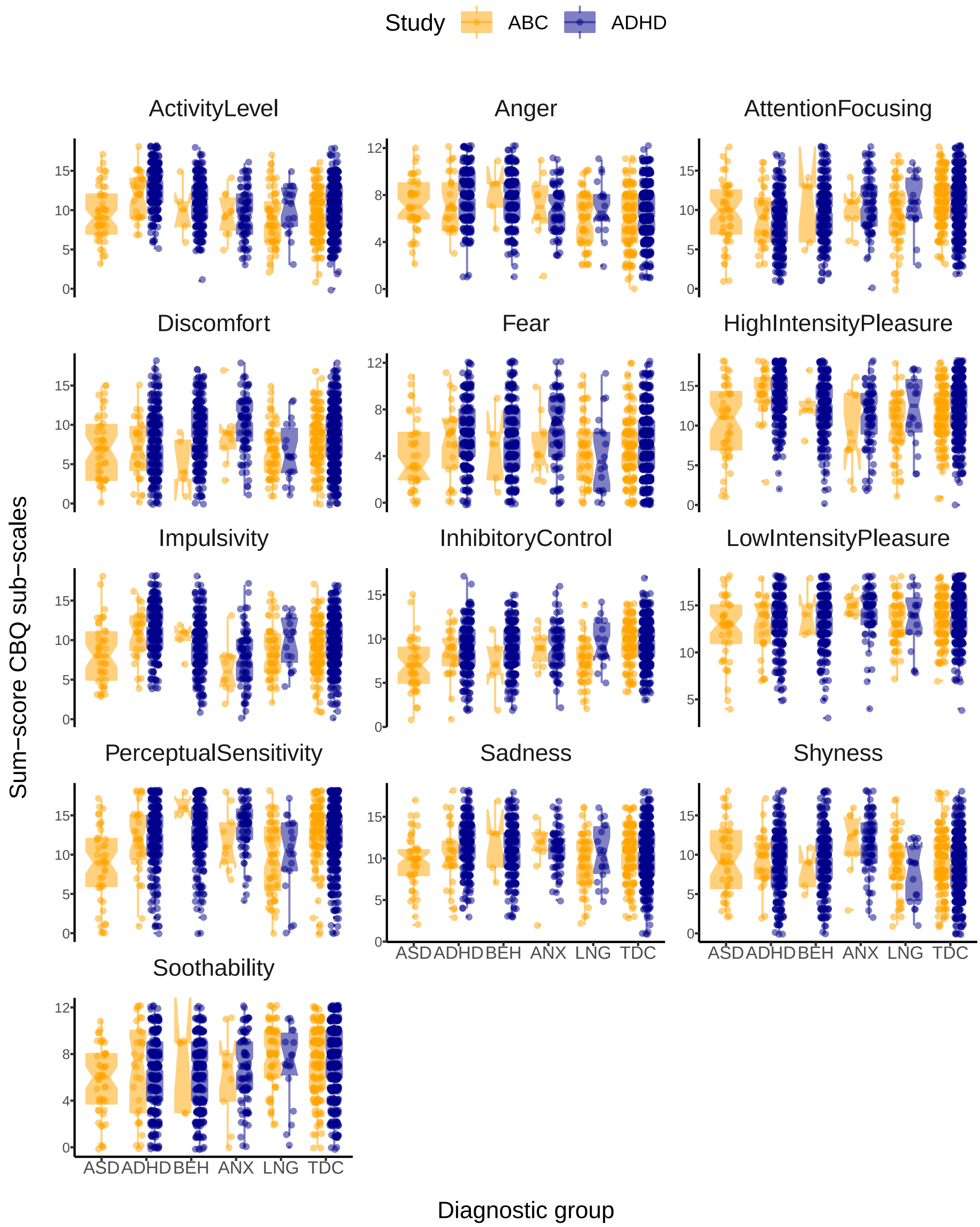


*Figure S17.* CBQ results in the ADHD and ABC studies, stratified by group.

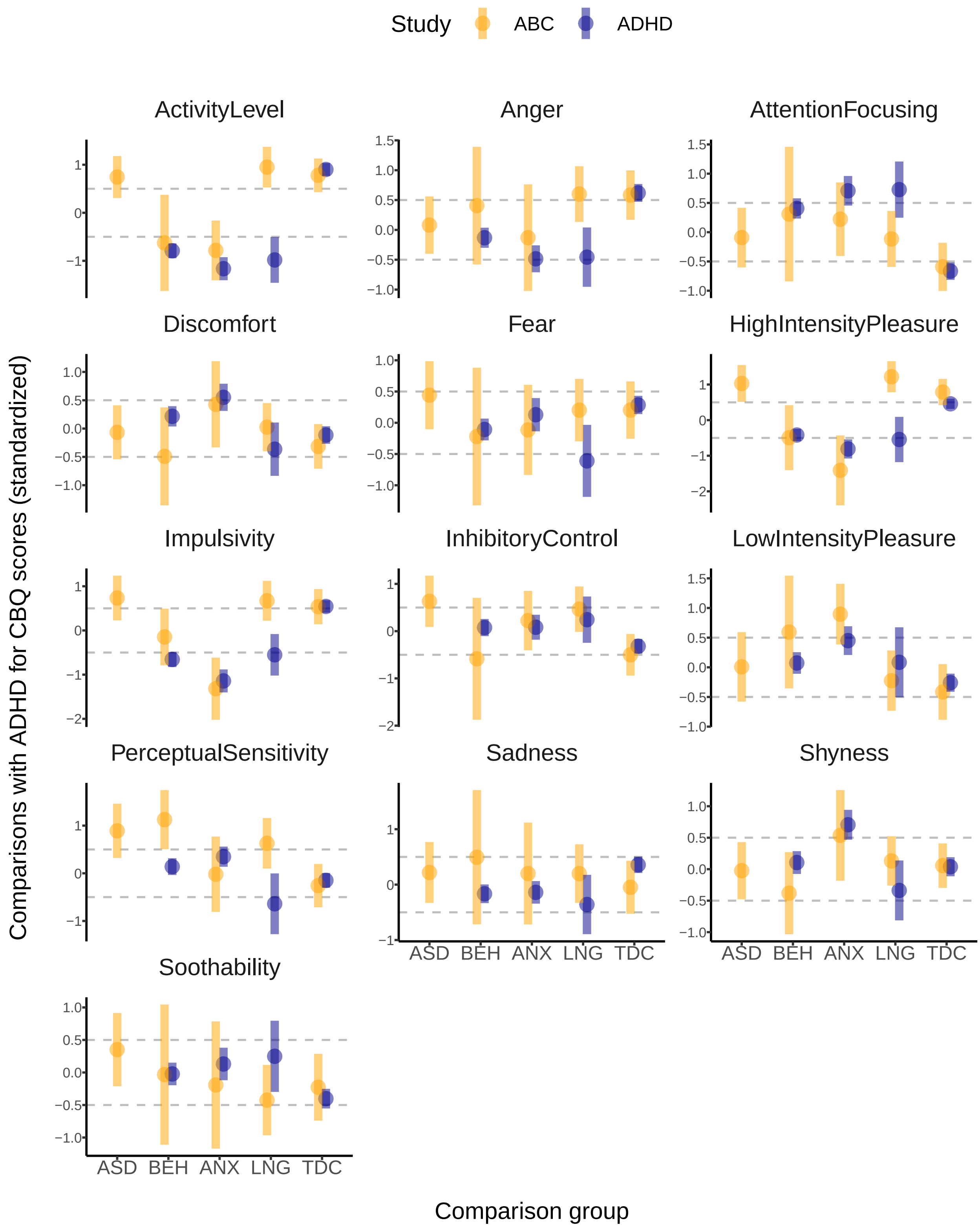


*Figure S18*. Comparison of children from ADHD and ABC Studies across CBQ results. All differences are based on scaled scores, whereby scaling was implemented by deviding the raw scores by the standardeviation of all scores (across sub-groups and studies).

Supplementary Material B

*

References

1. Stoltenberg C, Schjolberg S, Bresnahan M, Hornig M, Hirtz D, Dahl C, et al. The Autism Birth Cohort: a paradigm for gene-environment-timing research. Molecular psychiatry. 2010;15:676–80.

2. Egger HL, Erkanli A, Keeler G, Potts E, Walter BK, Angold A. Test-Retest Reliability of the Preschool Age Psychiatric Assessment (PAPA). Journal of the American Academy of Child and Adolescent Psychiatry. 2006;45:538–49.

3. Goodman R. The extended version of the Strengths and Difficulties Questionnaire as a guide to child psychiatric caseness and consequent burden. Journal of Child Psychology and Psychiatry, and Allied Disciplines. 1999;40:791–9.

4. Conners CK, Sitarenios G, Parker JDA, Epstein JN. The Revised Conners' Parent Rating Scale (CPRS-R): Factor Structure, Reliability, and Criterion Validity. Journal of Abnormal Child Psychology. 1998;26:257–68.

5. Sprafkin J, Volpe RJ, Gadow KD, Nolan EE, Kelly K. A DSM-IV-referenced screening instrument for preschool children: the Early Childhood Inventory-4. Journal of the American Academy of Child and Adolescent Psychiatry. 2002;41:604–12.

6. Gioia GA, Isquith PK, Retzlaff PD, Espy KA. Confirmatory factor analysis of the Behavior Rating Inventory of Executive Function (BRIEF) in a clinical sample. Child Neuropsychology: A Journal on Normal and Abnormal Development in Childhood and Adolescence. 2002;8:249–57.

7. Gioia GA, Isquith PK, Retzlaff PD, Espy KA. Behavior rating inventory of

executive functionpreschool version. Norwegian manual supplement. Lutz, FL: Psychological Assessment Resources; 2007.

8. Ireton H, Glascoe FP. Child Development Inventory Manual. Mn: Behavior Science Systems, Inc.; 1992.

9. Ireton H, Glascoe FP. Assessing children's development using parents' reports. The Child Development Inventory. Clinical Pediatrics. 1995;34:248–55.

10. Putnam SP, Rothbart MK. Development of short and very short forms of the Children's Behavior Questionnaire. Journal of Personality Assessment. 2006;87:102–12.

11. Buss AH, Plomin R. Temperament: Early Developing Personality Traits. Psychology Press; 1984.

12. Mathiesen KS, Tambs K. The EAS temperament questionnaire–factor structure, age trends, reliability, and stability in a Norwegian sample. Journal of Child Psychology and Psychiatry, and Allied Disciplines. 1999;40:431–9.

13. Coplan RJ, Rubin KH. Exploring and Assessing Nonsocial Play in the Preschool: The Development and Validation of the Preschool Play Behavior Scale. Social Development. 2001;7:72–91.

14. Skogan AH, Egeland J, Zeiner P, Øvergaard KR, Oerbeck B, Reichborn-Kjennerud T, et al. Factor structure of the Behavior Rating Inventory of Executive Functions (BRIEF-P) at age three years. Child Neuropsychology: A Journal on Normal and Abnormal Development in Childhood and Adolescence. 2016;22:472–92.

15. Korkman M, Kemp S, Kirk U. NEPSY: A Developmental Neuropsychological

Assessment. PsychCorp; 1998.

16. Korkman M, Kemp S, Kirk U. NEPSY. Handbok [Handbook]. Stockholm, Sweden: Psykologiforlaget AB; 2000.

17. Hughes C, Ensor R. Executive function and theory of mind in 2 year olds: a family affair? Developmental Neuropsychology. 2005;28:645–68.

18. Matthews CG, H. K. Wisconsin Motor Steadiness Battery. Administration Manual for Child Neuropsychology Battery. Madison, WI: Neuropsychological Lab, University of Wisconsin; 1978.

19. Kaplan E, Goodglass H, Weintraub S, Brand S. Boston Naming Test. Lea & Febiger; 1983.

20. Roid GH. Stanford Binet intelligence scales. 2003.

21. Campbell SB, Szumowski EK, Ewing LJ, Gluck DS, Breaux AM. A multidimensional assessment of parent-identified behavior problem toddlers. Journal of abnormal child psychology. 1982;10:569–91.

22. R Core Team. R: A language and environment for statistical computing. Vienna, Austria: R Foundation for Statistical Computing; 2017. https://www.R-project.org/.

23. Heinzen E, Sinnwell J, Atkinson E, Gunderson T, Dougherty G. Arsenal: An arsenal of 'r' functions for large-scale statistical summaries. 2018. https://CRAN.R-project.org/package=arsenal.

24. Bürkner P-C. brms: An R package for Bayesian multilevel models using Stan. Journal of Statistical Software. 2017;80:1–28.

25. Bürkner P-C. Advanced Bayesian multilevel modeling with the R package brms.

The R Journal. in press.

26. Fox J, Weisberg S. An R companion to applied regression. Third. Thousand Oaks CA: Sage; 2019. https://socialsciences.mcmaster.ca/jfox/Books/Companion/.

27. Fox J, Weisberg S, Price B. carData: Companion to applied regression data sets. 2018. https://CRAN.R-project.org/package=carData.

28. Dowle M, Srinivasan A. Data.table: Extension of ‘data.frame‘. 2018. https://CRAN.R-project.org/package=data.table.

29. Soetaert K. Diagram: Functions for visualising simple graphs (networks), plotting flow diagrams. 2017. https://CRAN.R-project.org/package=diagram.

30. Gohel D. Flextable: Functions for tabular reporting. 2019. https://CRAN.R-project.org/package=flextable.

31. Wickham H. ggplot2: Elegant graphics for data analysis. Springer-Verlag New York; 2016. http://ggplot2.org.

32. Gordon M. Gmisc: Descriptive statistics, transition plots, and more. 2018. https://CRAN.R-project.org/package=Gmisc.

33. Wickham H, Miller E. Haven: Import and export ’SPSS’, ’stata’ and ’SAS’ files. 2018. https://CRAN.R-project.org/package=haven.

34. Meredith M, Kruschke J. HDInterval: Highest (posterior) density intervals. 2018. https://CRAN.R-project.org/package=HDInterval.

35. Gordon M, Gragg S, Konings P. htmlTable: Advanced tables for markdown/HTML. 2018. https://CRAN.R-project.org/package=htmlTable.

36. Xie Y. Dynamic documents with R and knitr. 2nd edition. Boca Raton, Florida:

Chapman; Hall/CRC; 2015. https://yihui.name/knitr/.

37. Sarkar D. Lattice: Multivariate data visualization with r. New York: Springer; 2008. http://lmdvr.r-forge.r-project.org.

38. van Buuren S, Groothuis-Oudshoorn K. mice: Multivariate imputation by chained equations in r. Journal of Statistical Software. 2011;45:1–67. https://www.jstatsoft.org/v45/i03/.

39. Hallquist MN, Wiley JF. MplusAutomation: An R package for facilitating large-scale latent variable analyses in Mplus. Structural Equation Modeling. 2018;1–8. doi:10.1080/10705511.2017.1402334.

40. Gohel D. Officer: Manipulation of microsoft word and PowerPoint documents. 2019. https://CRAN.R-project.org/package=officer.

41. Aust F, Barth M. papaja: Create APA manuscripts with R Markdown. 2018. https://github.com/crsh/papaja.

42. J L. Plotrix: A package in the red light district of r. R-News. 2006;6:8–12.

43. Revelle W. Psych: Procedures for psychological, psychometric, and personality research. Evanston, Illinois: Northwestern University; 2018. https://CRAN.R-project.org/package=psych.

44. Neuwirth E. RColorBrewer: ColorBrewer palettes. 2014. https://CRAN.R-project.org/package=RColorBrewer.

45. Eddelbuettel D, François R. Rcpp: Seamless R and C++ integration. Journal of Statistical Software. 2011;40:1–8. doi:10.18637/jss.v040.i08.

46. Eddelbuettel D, Balamuta JJ. Extending extitR with extitC++: A Brief

Introduction to extitRcpp. PeerJ Preprints. 2017;5:e3188v1. doi:10.7287/peerj.preprints.3188v1.

47. Wickham H. Reshaping data with the reshape package. Journal of Statistical Software. 2007;21. http://www.jstatsoft.org/v21/i12/paper.

48. Stan Development Team. Rstanarm: Bayesian applied regression modeling via Stan. 2016. http://mc-stan.org/.

49. Soetaert K. Shape: Functions for plotting graphical shapes, colors. 2018. https://CRAN.R-project.org/package=shape.

50. Lüdecke D. sjPlot: Data visualization for statistics in social science. 2019. doi:10.5281/zenodo.1308157.

51. Stan Development Team. StanHeaders: Headers for the R interface to Stan. 2016. http://mc-stan.org/.

52. Gagolewski M. R package stringi: Character string processing facilities. 2018. http://www.gagolewski.com/software/stringi/.

53. Wickham H. Stringr: Simple, consistent wrappers for common string operations. 2018. https://CRAN.R-project.org/package=stringr.

54. Yoshida K, Bohn. J. Tableone: Create 'table 1' to describe baseline characteristics. 2018. https://CRAN.R-project.org/package=tableone.

55. Muthen LK, Muthen BO. Mplus User's Guide. Eighth Edition. Los Angeles, CA: Muthen & Muthen; 2017.

56. Rasch G. Probabilistic models for some intelligence and attainment tests. MESA Press; 1960.

57. Furr DC. Edstan: Stan models for item response theory. 2017. https://CRAN.R-project.org/package=edstan.

58. Asparouhov T, Muthen B. Exploratory Structural Equation Modeling. Structural equation modeling: a multidisciplinary journal. 2009;16:397–438.

59. Bürkner P-C. brms: An R package for Bayesian multilevel models using Stan. Journal of Statistical Software. 2017;80:1–28.

60. Carpenter B, Gelman A, Hoffman M, Lee D, Goodrich B, Betancourt M, et al. Stan: A Probabilistic Programming Language. Journal of statistical software. 2017;76:1–32.
